# Experiences and Challenges of Mothers in Caring for Infants with Delayed Developmental Milestones: A Case of Dodoma Region, Tanzania

**DOI:** 10.24248/eahrj.v8i1.757

**Published:** 2024-03-28

**Authors:** Helena Marco Gemuhay, Saada Ali, Stephen Kibusi

**Affiliations:** a Department of Clinical Nursing, School of Nursing and Public Health; b Department of Public Health, School of Nursing and Public Health, University of Dodoma, Dodoma, Tanzania

## Abstract

**Background:**

Delayed Developmental Milestones is a physical disability affecting the child development, occurs when the child fails to attain normal milestones compared to other children. Globally, 180-200 million infants have signs of developmental delay, and 86% are from developing countries. In Dar es Salaam, proportion of children with cognitive delay is 12.3%.

**Objectives:**

This study explored the experiences and challenges of mothers in caring for infants with delayed developmental milestones.

**Methods:**

In-depth interviews with ten mothers explored their experiences and identified challenges they encountered while caring for infants with delayed developmental milestones. NVIVO plus software was used for content analysis.

**Results:**

Results showed that parents raising infants with delayed developmental milestones had negative experiences on the cause and types of delayed milestones. They lost hope. They were facing different challenges like lack of specialists, conflict in marriage and stigma.

**Conclusion:**

Mothers of infants with delayed developmental milestones had negative experiences about this problem and they face many challenges in caring the infants.

## BACKGROUND

Delayed Developmental Milestones (DDM) is a physical disability usually appearing in childhood and affecting the child development. It occurs when the child fails to attain developmental milestones compared to peers from the same population.^1^ This condition manifests itself in various domains such as the sensory, motor, language/communication and social personal.^2^ Holding the head steadily unsupported at 3 months, rolling over at 6 months, crawling at 9 months, walking while holding on furniture at 12 months, walking without holding furniture at 18 months, and talking at 24 months are milestone domains considered normal for an infant.^3, 5^ Infants get diagnosed with DDM if they do not achieve any of the milestone domains at a specific time.^6^ If only one domain is delayed, it is called specific delay, and if multiple domains are delayed, it is called global delayed development (GDD).^7^

Delayed developmental milestones account for more morbidity across the life span than any other chronic condition.^8,9^ Globally, every year 180-200 million of infants present with features of developmental delay, 86% of these infants with DDM and 4% without DDM are from developing countries while 8% with DDM and 2% without DDM are from developed countries.^10^ The incidence of cognitive delay of 12.3%^11^ has been reported by a study which was conducted in Dar es Salaam.

A study conducted in India, showed some challenges such as poor utilisation of health care facility in infants with specific delayed milestone and negative experiences about DDM.^12^ The situation is worse for the infants with global developmental delays where multiple interventions are required.^12^ A study conducted in Namibia observed negative experiences among community members, as a result of factors such as community attitudes, social cultural beliefs as well as institutional challenges and evil spirits which made mothers to be accused by their husbands.^13^

Inadequate knowledge, lack of health care facilities and insufficient resources for caring of infants with DDM were associated with poor socio-economic status.^14^ Parents of infants with DDM come across challenges at work, at home, because of failure to cope with the infant's disability and financial challenges. In most cases, parents are unsure of what was expected of them in making educational decisions on behalf of their infants and awareness on coping mechanisms is low,^15^ as a results they undergo psychosocial problems which affect marital relationship due to stress, guilt and accusation.^16^

Inadequate support from the spouse/family, and the community are among the challenges faced by parents of infants with DDM.^17^ This situation creates psychological torture and results in fear of being rejected by the families/friends and lack of financial support.^18^ Infants with DDM require special care, and sometimes it is difficult to get insurance coverage for health care service.^19^ In Tanzania, there is a paucity of evidence on the mothers' experiences and challenges encountered in the process of caring for infants with disabilities. The objectives of this study were to explore mothers' experiences and identify challenges in caring for infants with delayed developmental milestones.

## METHODOLOGY

### Study Design and Setting

The study applied descriptive phenomenology design which was conducted on mothers of infants with DDM by using in-depth interviews. This study was conducted at Dodoma Regional Referral Hospital, in Dodoma, Tanzania. The hospital is a referral and teaching hospital that serves over 2 million residents of Dodoma Region as well as other people from neighboring districts of Kiteto (Manyara Region), Manyoni (Singida Region) and Gairo (Morogoro Region). It is the largest hospital located in the Dodoma Municipality with bed capacity of 420. The average number of patients who are admitted every day is 42 and with 83% being the average bed occupancy rate.^20^

### Study Population

The study population included mothers of infants with DDM and who consented to participate in the study.

### Sample Size and Sampling

The sample size of 10 mothers is in line with the recommended sample size for qualitative studies which may range between 5 to 50 depending on the saturation point.^21,22^ Convenience sampling was used to select mothers in the study.^21^ Ten to 15 infants a day were brought to the hospital, and mothers were chosen based on their availability during the data collection period. Mothers of infants with DDM who visited the health facility for treatment and rehabilitation services at physiotherapy department were eligible for the study.

### Data Collection Method

Data collection interviews were conducted between July and August 2022. The principal investigator (PI) was assisted by research assistants who were diploma and degree holders. They carefully noted and correctly recorded the information given by the mothers. The researcher and the research assistants created social environment for the study participants so that they could be trusted. Research assistants were taught how to behave during interview like being natural and presentable, briefly taking the note using audio tape for recording, and adhering to the ethical issues. For almost 3 weeks, the interviews were conducted on Monday and Friday when the DDM clinic was in operation. The interview schedule was based on the time mothers arrived at hospital and their date of appointment. The interview was carried out in Swahili and lasted 30 to 40 minutes.

### The Tool for Data Collection

The tool consisted of open-ended questions which guided the interviewer to explore mothers' experiences and identify challenges in caring infants with DDM. This tool was developed by the PI based on the reviewed literature and consisted of 15 questions.

### Ethical Considerations

Ethical approval to conduct this study was obtained from the University of Dodoma Ethical Review Board with a Reference number (MA.84/261/02/35). The permission to conduct the study was given by the Dodoma Regional Referral Hospital. Before conducting the interview, the researchers obtained informed consent verbally and in written form, the privacy was assured for the participants for them to be free to provide information without any restraint during in-depth interview.

### Data Analysis

Data were analysed using content analysis. All audio taped information was transcribed verbatim. The texts were broken down into small manageable codes for analysis,^22^ the codes were generated, and sub themes and themes developed.

Eight steps cited on content analysis were used to guide in the analysis of the data.^23^ These steps include (1) preparation of data (2) defining the themes of analysis (3) developing categories and coding themes (4) Pre-testing the coding scheme on sample (5) coding all the texts (6) assessing the consistency of employed coding (7) drawing inferences on the basis of coding or themes and (8) presentation of results.

Trustworthiness of the qualitative content analysis was observed by using terms such as credibility, dependability, conformability and transferability.^24^ Credibility was achieved through the use of strategies including saturation of data, transparency, consistency during data processing, and providing participants sufficient time to provide in-depth information.

Dependability was attained by using technology, such as data analysis software, pilot testing, systematic data collection and proper documentation.^25^ Confirmability was achieved by demonstrating that the findings were linked to the conclusions. Audio recording and fields notes were taken during data collection process to ensure that all important data were not missed. Its relevance to application is similar to credibility, where confirmability has particular implications for studies that give policy recommendations.^26^ Transferability is established by providing readers with evidence that the research study's results could be applicable to other contexts, situations, times, and populations.^27^

Content analysis was used to analyse the mothers' experiences and challenges in caring for their infants with DDM. Familiarizing, transcribing, sorting and coding of the data were done by researchers for them to be aware of the responses obtained from the study participants. The data with similar meaning were assembled together into themes and the emerging themes are discussed and supported by related literature.

## RESULTS

### Background Characteristics of the Study Participants

The results from the study participants, who reported their experiences and challenges when caring for infants with DDM, are presented in this section. Ten study participants were involved, and their demographic characteristics included the mother's age, mode of delivery, the child's sex, place of residence, and the type of DDM (Table 1).

**TABLE 1:** Social Demographic Characteristics of Study Participants

Name	Age of Mother	Age of child (Months)	Delivery Mode	Domicile	Hospital	Language	Last Date visit of Hospital	Sex	DDM type
No4	38	12	Normal	Dodoma	DRRH	Kaguru	30/12/2021	Male	Motor
No1	30	12	Caesarean section	Nkuhungu	Makole	Rangi	30/1/2022	Male	Motor
No10	30	12	Normal	Dodoma	DRRH	Gogo	7/1/2021	Male	Language, eating & motor
No2	25	12	Normal	Dodoma	DRRH	Nyakyusa	Not remembered	Female	Motor
No3	25	12	Normal	Dodoma	DRRH	Haya	First time	Male	Motor
No5	31	12	Normal	Dodoma	DRRH	Gogo	30/12/22	Female	Motor
No6	28	9	Normal	Bahi	Bahi	Gogo	21/6/2021	Female	Motor
No7	29	12	Normal	Dodoma Area A	DRRH	Gogo	7/2/2022	Male	Motor, personal social
No8	29	9	Normal	Bahi	DRRH	Gogo	4/2/2022	Male	Motor
No9	27	12	Normal	Dodoma	DRRH	Rangi	Unassigned	Female	Language, eating & motor

**Source:** Field Data 2022

### Mothers' Experiences and Challenges Encountered in Caring for Infants with DDM

Three main themes emerging from the data included (1) Experiences, (2) Challenges and (3) Child development activities.

Table 2 shows the themes and their sub-themes. Experiences were further divided into: understanding or meaning, types, causes, treatment, exercises, hope for cure, and feelings. Challenges were categorized into lack of spouse involvement in child care, treatment costs, lack of specialists, conflicts in marriage, accusations and conflicts with relatives and neighbourhood challenges. Child development activities were categorized into three parts: playing toys, storytelling and doing child favourite activities.

**TABLE 2:** Generated Codes, Subthemes and Themes on the Description of Experiences and Challenges of Mothers on Caring DDM Infants. (N=10)

Meaningfully sentence	Codes	Subthemes	Themes
DDM is health care problem like other diseases, my child has difficulty eating, cannot sit, no neck control, cannot crawn or stand & my child changes colour	-Believes on concept of DDM -I don't know Growth challenge -Mismanagement during labour and delivery -She had labour pain for long time -Meconium aspiration -Lack of oxygen in the brain -She mates an ugly person during pregnancy that's why she delivered an ugly child -Brain dysfunction	Understanding of DDM	Experiences of mothers in caring infants with DDM.
	-Convulsion -High temperature -Neonatal jaundice -Meningitis -Malaria -Marasmus kwashiorkor -Anaemia	Believes on causes of DDM	
	Motor, personal social & eating difficulty	Believes on types of DDM	
	-During delivery they were not given adequate care -Carelessness by health care providers -Pronged labour & meconium aspiration	Negligence	
	-Exercises -Medication -Operation -Surgery	Treatment service experience	
Marriage has conflicts because of this child. The treatment cost is expensive & the father sees that having a child like this is a curse in the family, Availability of service is a challenge; sometimes there is no specialist to exercise her, the father has problem with the mother concerning the child. He believed that the child was born like this because the mother has problem. He believes the child disability is caused by the mother.	-Parents lost hope	Hope & despair for a cure	Challenges of mothers in caring infants with DDM
	-Lack of medical specialist -Spouse involvement in child treatment -Expensive treatment -Marriage is not at peace	Lack of specialist Spouse involvement Treatment costs Marriage in conflicts	
	-Mother is accused -Father is accused -Witch craft -Health care workers -No one is accused	Accusation	
	-Call it disabled -Devil and evil spirit -No treatment -Child is a gift from God -Unknown	Neighbours' conflicts	
He plays with his hands; I don't give him dolls because they can make him have unusual spirit & he sucks his fingers & watches TV.	Playing games & toys Involvement of family members in playing with a child	Play with child	Child development activities
	Storytelling, smiling, singing & standing the child No activities were done by their children Children were able to play with their hands, can stand and play with various objectives.	Storytelling & standing Child favourite & most minor activities	

### Theme 1: Mothers' Experiences in Caring for Infants with DDM

Experiences refer to observing or undergoing something that generally occurs in the course of time, to learn from experience. Mothers' experiences of DDM were further categorized into three subthemes: understanding DDM in terms of causes, types and treatment.

### Mothers' Understanding or Beliefs on DDM

Half of study participants who had spontaneous vaginal delivery said that DDM is a health problem like other diseases. Of those, three were aged between 20-29 years, and two were aged 30 - 39 years. A typical response was, *“This is a health problem like other health problems,”* Number 9 (Age 27, child age in months 12). Another similar response was, *“I believe it is a health problem,”* Number 6 (Age 28, Child age in months 9), Delivery mode: Spontaneous Vaginal Delivery). However, not everyone had exactly a similar perception. For example, one study participants reported that DDM was a growth challenge. She said, *“She has a growth challenge. She is growing slowly,”* Number 5 (Age 31, Child age in months 12). The responses indicate a common perception and understanding of the disease with slightly different perspectives-one emphasized on slow in the growth of the infant.

### Experienced DDM Type

Delay in motor domains was reported by all study participants. Delay in language domain was reported by 3 participants and delay in personal social was observed in 1 participant.

### Beliefs on the Causes of DDM

Repeated reading of the datasets found that mothers believed that DDM was also associated with convulsion, anaemia, high temperature, malaria, marasmic kwashiorkor, meningitis and neonatal jaundice. However, convulsion and high temperature were more reported by mothers than other causes.

### Negligence

Four out of 10 interviewees expressed that DDM for their infants was caused by carelessness of healthcare workers during the delivery. The negligence involved “wrongly pulling out the infant, prolonged labour and the infant drinking “dirty” water during delivery”. The content analysis found that during delivery, the mothers believed that they were not given adequate delivery services. On this regard, the study participants said:

*“My child was wrongly pulled when I was giving birth. Prolonged labour has made the child to be like this,” Number 2 (Age 25, Child age 12 months).*

*“This is a health problem. I believe the child drank a lot of dirty water during delivery,” Number 3 (Age 25, Child age in months: 12).*

Furthermore, participants believed their infants lacked oxygen due to carelessness during delivery. Babies lacked oxygen which caused DDM in their infancy. Moreover, doctors/nurses, severe bleeding, and yellow fever were also said to have contributed to the problem. The responses indicate that DDM could have been avoided by providing good delivery services at the clinic. On the same regard, 2 study participants reported that:

*“No, this is due to the doctors' and nurses' negligence. Meaning, that oxygen does not enter the brain upon birth. “Lack of oxygen in the brain was caused by the doctor's negligence,” Number 7 (Age, 29, Child age in months: 12).*

*“It was caused by negligence by nurses and doctors at the hospital. These factors caused “severe bleeding and the child got yellow fever,” Number 8 (Age, 29, Child age in months; 8).*

More details about causes and types of DDM are found in Table 3.

**TABLE 3:** Causes of DDM vs Types of Delivery Mode

Causes of DDM	Mother Delivery Mode	
	Spontaneous Vaginal Delivery	Caesarean Section
Neonatal jaundice	2 e.g., My child changed colour, became yellowish. Number 3	0
Meningitis	1 e.g., She got Meningitis	0
Marasmic kwashiorkor	3 e.g., My child has malnutrition, she also had seizure	0
Malaria	1 e.g., My child suffered from malaria, malnutrition, her temperature rose and her	0
High temperature	3 e.g., His temperature rose early and he got convulsion	0
Convulsion	6 e.g., immediately after she has a convulsion	0
Anaemia	1	0

**Source:** Field Data 2022

### Treatment for DDM

Eight out of 10 study participants reported that their infants received outstanding treatment services of various kinds. The treatment involved medications, physical exercises and operations. Medications were reported by parents who believed that DDM was a disease like any other illness and therefore, they insisted on attending health facilities for treatment. However, not every participant agreed that all DDM victims were getting treatment; some were not receiving any treatment. Two study participants reported not getting treatment, and the other expressed difficulties in accessing treatment. On this, participants said:

*“The child is undergoing treatment. The father has no problem. He insists we follow the treatment,” Number 4 (Age 38, Child age in months: 12).*

*“The child is under medication,” Number 3 (Age 25, Child age in months: 12).*

*“The child is not on medication today. It is the first time I have brought her to hospital. She is coughing. She has not got any medication for this disease.” “Number 6 (Age 28, Child age in months: 9).*

*“Availability of service is a challenge; sometimes there is no specialist to involve my child in exercises.” Number 2 (Age 25, Child age in months).*

These responses imply that the study participants indicated good understanding of the problem and the need to take infants with DDM for treatment.

### Exercises

Most of mothers reported their infants were undertaking physiotherapy as part of the treatment programme. However, some infants with DDM were also receiving treatment related to other illness which is thought to be the cause of the DDM. On this, the study participants said:

*“Yes, we take him for exercises twice a week*. *It should be noted that the treatment for the DDM is exercise but convulsion is treated by medication,”* Number 7).

*“I bring him for exercises every Monday and Friday,”* Number 8 (Age, 29, Child age in months 9).

Yet, there was little understanding of whether exercises were a kind of treatment or not.

### Theme two: Challenges in caring for infants with DDM

**FIGURE 1:**
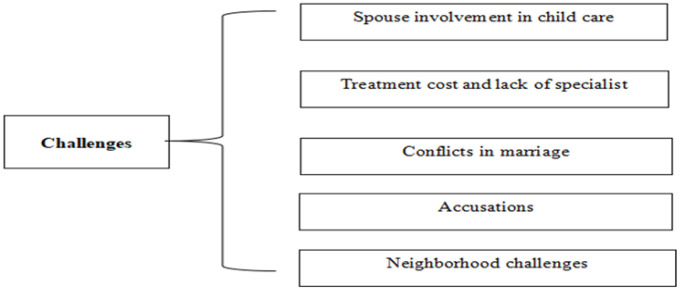
Types of Challenges Facing Mothers of Children with DDM

Source: Field data 2022.

Hope and despair for a cure, spouse involvement in child care, treatment costs and lack of specialists, conflict in marriage, accusations and stigmatization were identified as challenges related to the care of infants with DDM (Figure 1).

### Hope and despair for a cure

Interviewees had mixed feelings about the treatment and cure of their babies with DDM. Five out of 10 interviewees expressed that their infants would be cured as they continued receiving treatment and that DDM was just a regular health challenge that was similar to other diseases. Therefore, interviewees who hoped for a cure insisted on clinic attendance for the babies' treatment and other health services. But not every interviewee had similar perceptions. On the contrary, two study participants said:

*“The father has lost hope on the treatment. “He does not help in any way,” Number 2 (Age 25, Child age in months; 12).*

*“He says the child cannot be cured. Family and relatives do not give any help. My husband does not want her. Although he has not said it; I see this through his actions. He shows that he does not like her because she is disabled”. Number 2 (Age 25, Child age in months; 12).*

The responses above indicate hope and despair. As the findings suggest, some parents hoped that their infants with DDM would be cured, but others had lost hope completely. They believed that their babies were disabled and thus could not be treated and cured. Furthermore, as the findings revealed that, there is tension and weird situations about the baby with DDM in the family about hope for treatment. The stress happens when one parent believes that the baby can be cured and another parent thinks the child cannot be treated and cured. The anxiety and different views affect the treatment the baby receive. Where the father does not believe that the baby would be cured, he would not support the mother in taking care of the baby including meeting the treatment costs.

*“This is a health problem; I believe one day my child will be cured. If the child exercises, she will be cured. But as it is the case, the father has lost hope on the treatment. He does not help in anyway. He says the child cannot be cured. Family and relatives do not give any help. My husband does not want this child, although he has not said it but I see his actions. He shows that he does not like her because she is disabled”. Number 2 (Age 25, Child age in months: 12)*

Moreover, it should be noted that it is a huge dilemma when the mother loses hope that the baby cannot be cured. This dilemma leads to a situation in which improper care and treatment is given to the baby by the mother. The mother is no longer motivated to take the baby to the physiotherapist. A typical response on this was:

*“The father has no any help. He insists we follow the treatment. Mother, I have lost hope,” Number 4 (Age 38, Child age in months 12).*

A perplexing response was:

*“I cannot say anything, as the child is a gift from God. The child will recover somehow,” Number 1 (Age 30, Child age in months: 12).*

**FIGURE 2:**
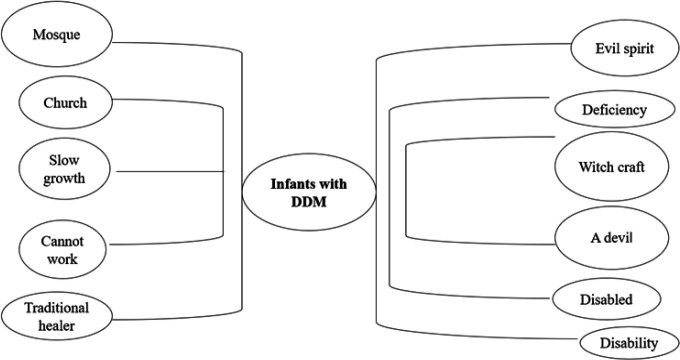
Words Used by Community Members to Describe a Child with DDM and the Treatment Places

Source: Field data 2022.

The responses above indicate that the participants would further insist on more treatment and believe the child would be cured. However, as it has been revealed in this example, the mother has completely given up (Figure 2).

### Lack of Specialists

Lack of specialists was found to be one of the challenges parents were facing when taking their infants with DDM to hospitals. It was noted that the hospitals were not equipped with doctors and other health care providers specialized to care for infants with DDM and could provide the needed treatments and exercises. It was found that therapy and exercises were sometimes offered by health workers who did not have any expertise. It was noted that the medical workers were demoralizing the parents who were visiting the centres for specialized treatment. In the worst scenario, they end up getting unintended services. A typical response with regard to this situation was:

*“Availability of service is a challenge; sometimes there is no specialist to attend her.” Number 2 (Age 25, Child age in months; 12).*

The response indicates discouragement after realizing that the parent is much better at helping the infant than the health workers at the clinic where the parent went for treatment.

### Accusation (Who is the source of DDM is it mother, father or health care provider)

There were contradictory views on the source of the DDM among the study participants. Two out of 10 participants said that husbands were accusing their wives (mothers of infants with DDM) to be the source of DDM. A typical response was:

*“The father says the child is like this because of the mother” Number 4 (Age 38, Child age in months; 12).*

*“The father has a problem with the mother concerning the child. He believes the child was born like this because the mother has a problem. He believes the child's disability was caused by the mother. The child's mother, “Number 2 (Age 25, Child age in months 12), “the child was born like this because the father has bad legs” Number 2 (Age 25, Child age in months: 12)*

However, not all of the respondents were sharing the same views. Two other participants expressed that negligence of health providers were causing DDM when attending mothers during delivery. It was alleged that health workers' negligence resulted in poor delivery services which results in babies being born with DDM. An exemplary response on this were:

*“The doctors are the cause of DDM due to their negligence. For my case, I gave birth to a child like this, and they allowed me to give birth normally despite knowing the baby had more weight than I could manage to deliver normally “Number 7 (Age, 29, Child age in months 12).*

*“Yes, it is a health problem, but it was caused by nurses' and doctors' negligence in the hospital. The healthcare workers were negligent when I was giving birth which led to my child's problem. Number 8 (Age, 29, Child age in months; 8).*

However, three other participants did not believe that healthcare workers' negligence being the cause of DDM.

*“Nobody is to be accused for DDM in infants”*.

Yet again, another participant said that:

*“DDM occur due to witchcraft.”*

These responses indicate disagreement among the participants about who is responsible for DDM in the community. Furthermore, the responses indicate lack of knowledge and understanding about the cause of the problem.

### Conflicts in Marriage

The findings show that there were problems in the families which led to lack of peace because the families were using a lot of money to treat their babies with DDM which put a burden on the families' resources thus fuelling misunderstanding and blames. Furthermore, it was reported that treatment costs were also causing more trouble in the marriages. A participant said:

*“My marriage has conflict because of this child. This is largely because the high treatment cost and the father believes that having a child like this is a curse in the family.” “Number 9 (Age 27, Child age in months; 12).*

Seven out of 10 study participants said that there was no problem with the marriage because of DDM. This is to say that the husband and wife would cooperate to take care of the infant. On this particular regard, participants said:

*“The marriage is stable but only that the costs of treating the child with DDM are high.” Number 9 (Age 27, Child age in months 12).*

*“The father and I do not have a problem concerning the child, my marriage is peaceful.” Number 4 (Age 38, Child age in months: 12).*

*“There is no problem to the father and I, we cooperate in helping the child,” Number 10 (Age 30, child age in months: 12).*

On the other hand, 3 out of the 10 participants stated that there were problems in the marriage because of having a child with DDM. On this, the participants said:

*“The father has lost hope in the treatment. He does not help in any way. He says the child cannot be cured. Relatives do not give any help,” Number 2 (Age 25, Child age in months: 12).*

### Treatment Cost

Treatment costs are high and parents with no health insurance were obliged to pay a certain amount of money. On this, the participants said:

*“The problem we get is that we use a lot of money to treat the child, for us with no insurance, we open a file for 15,000 every time we go for Clinic, we use a lot of money for the treatment,” Number 7 (Age, 29, Child age in months: 12).*

The response above means that the costs of treatment were intolerable to some families. This might be a reason for discontinuing the therapy and abandoning the baby with DDM.

### Stigmatisation

Stigmatisation is another challenge experienced by parents of infants with DDM. The challenge originates from the community members include neighbours who use different words that suggested stigma when referring to infants with DDM. Such words were creating psychological trauma to parents of infants with DDM. The stigmatization make parents feel that giving birth to an infant with DDM was something to be ashamed of. Typical responses on this were:

*(From Neighbours) “The child is disabled. He cannot walk. He should go to church or mosque to be prayed for. He should see a traditional healer because an evil spirit, or the devil, that missed harming the mother during pregnancy went to the child. Neighbours talk poorly of the child “Number 3 (Age 25, Child age in months: 12, Delivery mode: SVD).*

*“I do not know; some say he won't be cured; everyone is saying their own thing. They call such infants disabled,” Number 1 (Age 30, Child age in months 12, Delivery mode: Operation).*

These responses indicate presence of stigmatization from the community members. Besides stigmatization, qualitative data indicate that misconceptions about infants with DDM were associated with evils. Infants with DDM were called devils, evil spirits and kind of some witchcraft. Linking infants with devils was making the community suggest that these infants should be taken to the church or mosque for prayers. The community was also suggesting to parents that they seek treatment from traditional healers Figure 2 highlights the perceptions and words used to refer to infants with DDM.

### Theme thee: Experience on Child Development Activities

Interviewees reported 3 child development activities they were often doing to their infants with DDM. The activities included talking and singing, standing and playing games. Talking and singing were the activities which were found to be preferred by infants. Furthermore, it was noted that other parents were going beyond singing. It was found that they were dancing for their infants, something which seemed to engage the kids mentally and therefore helping them in their development. The typical responses were:

*“I talk and laugh with him, and he laughs back,” Number 1 (Age 30, Child age in months: 12).* Another similar response was, *“I try to talk to him and sing to him songs,” Number 10 (Age 30, Child age in months 12).*

*“She enjoys playing with toys and me singing for her,” Number 5 (Age 31, Child age in months: 12). “I sing and dance for her, “Number 9 (Age 27, Child age in months 12).*

### Playing Games

Parents had a tendency of playing games with their infants, the games included were; toys, the parent physically playing with their kids, and doing exercises. The study also found that the child's dad, the mother, relatives, house helpers or maids and other kids were involved in playing with the child. On this regard, study participants said:

*“Yes, I also play with him sometimes and make him do exercises to straighten his legs,” Number 7 (Age, 29, Child age in months; 12, Delivery mode: SVD)*.

*“Just playing with the child,” Number 6 (Age 28, Child age in months; 9, Delivery mode*: SVD).

*“He only plays with his brother,” Number 10 (Age 30, Child age in month 12, Delivery mode: SVD).*

*“She plays with her dad or sister,” Number 9 (Age 27, Child age in months: 12, Delivery mode: SVD).*

However, in some families, the mother was the only person who played with the child, and in other families, the task was left with the house helper, popularly known as house girl in Tanzania.

### Storytelling and Standing

The analysis found that storytelling and standing were not being used as development activities. Six out of the 10 participants said that: they were not using storytelling and the remaining participants did not report whether they were using it or not. Similarly, it was found that standing was less frequently being used as part of the development activities.

### Child's Favourite and Most Minor Activities

Based on the findings, 5 out of 10 interviewees do not know their child's favourite and most minor activities, because most of the time, the child is lying down. However, other parents reported different activities such as playing with hands, standing up and playing with various physical objects. For instance, the study participants said:

*“He cannot play, because he just lies down.” Number 9 (Age 27, Child age in months; 12, Delivery mode: SVD).*

*“I do not know what to do with her due to her condition,” The interviewee number 10 (Age 30, Child age in months: 12, Delivery mode: SVD) said.*

*“He plays with his hands, I don't give him dolls because they can make him have unusual spirits, and he sucks his fingers and watches TV, “Number 1 (Age 30, Child age in months 12)*

*“She likes when I stand her up although she cannot stand on her own, “Number 2 (Age 25, Child age in months; 12)*

### Mixed feelings for Giving Birth to Child with DDM

Eight out of 10 interviewees expressed that they did not regret giving birth to a child with DDM because the child was a gift from God. The child is God's creation. A typical response was:

*“I do not feel guilty having this child because it is a gift from God,” Number 2 (Age 25, Child age in months: 12). A similar response was, “I do not feel guilty having this child because I am given by God,” Number 5 (Age 31, Child age in months 12).*

Yet, two other participants expressed different views:

*I feel guilty and regret having a child with DDM. The participant number 7 (Age, 29, Child age in months 12) said, “Yes I regret because most of the time I spend caring for the child.” The second participant disclosed, “I regret because the child has delayed walking,” Number 9 (Age 27, Child age in months: 12).*

**FIGURE 3:**
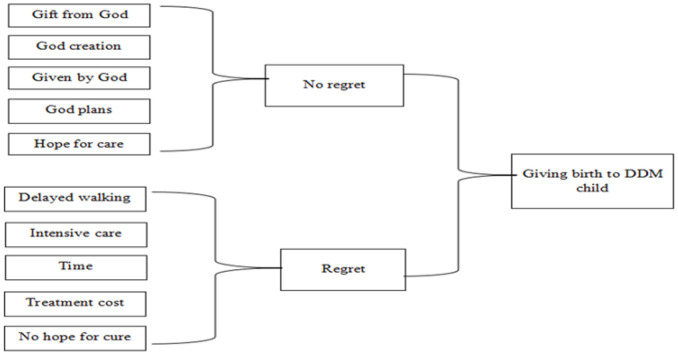
Mixed Feelings for Having Given Birth

Source: Field data 2022.

What do the responses mean? The responses indicate disagreement. Although 8 participants showed they did not feel guilty, the other 2 indicated very deep worries and regretted having given birth to the child with DDM (Figure 3)

## DISCUSSION

### Mothers' Experience and Challenges in Caring for Infants with DDM

### Theme 1: Mothers' Experiences in Caring for Infants with DDM

The current study showed negative experiences of parents in raising their infants with DDM, their beliefs on causes, types and treatment were not clear. It was found that, majority of the participants believed that the cause of DDM could be high temperature, convulsion, neonatal jaundice and meningitis. The similar study suggest that neonatal sepsis/meningitis and convulsions also showed a significant association with DDM.^28,29,30^ This is because pregnancy and delivery mode exposes the child to many pathogens through the vagina, ruptured amniotic membranes or by neonatal skin contacts via blood stream to central nervous system.

On the other hand, study participants mentioned witchcraft, evil spirits, and accusation for example, the father accused his wife as the source of DDM to child, this concurs with the study done in Kenya which revealed that people engage in gossip since DDM might have a number of challenging causes, such as cultural beliefs and witchcraft, when a parents gets a child with DDM, people may think of witchcraft.^31^ Generally, DDM can occur due to medical reasons.^1^ The observation from previous studies portrays that the prominent causes of DDM in African countries are the same and can be prevented or minimized through comprehensive early intervention for child bearing mothers and infants before 3 years of age. However, community education can minimize misconceptions and wrong African beliefs about DDM.

Shortage of health facilities offering treatment for disabled infants. It seems that in other places there are no health facilities that provided treatment for these infants. These findings are supported by Alaee FS and Kermanshahi.^32^ They reported that the main problems are the lack of health care facilities and the limitation of parents' social interactions with their children who have DDM, they must travel far away to get health care services. The other similar study observed that DDM can be linked to poverty and insufficient health care services.^33^ Lack of qualified healthcare providers and DDM-friendly health care facilities can significantly affect the quality of the services, which may discourage parents from utilising those services.

### Challenges of Mothers in Caring for DDM Infants

Limited treatment services due lack of specialists was reported by mothers as one of the challenges, they were facing. They said that they were coming from far away and finding that there was no expert to treat their infants, they reported that the kind of exercises which are given to their infants, they can do at home. The similar study which concur with the current study observed that, inadequate health care services was a leading cause of cognitive delayed developmental milestones.^11^ Due to a lack of specialists, infants with DDM might not always have access to the various therapies that they need, such as speech therapy or physical therapy.

The study also observed lack of spouse involvement in child care. On this; mothers reported that due to disability, their husbands were desperate so they did not help anything about child. Similarly, studies have shown that spouses do not take part in child care, mothers reported that their spouses do not provide any help with child care because of the DDM.^34^ Gender roles and expectations within the family can be influenced by cultural and societal standards, there may be a particular expectation in various cultures about the caring roles.

Parents encountered financial problems in raising infants who were suffering from DDM and the treatment costs exceeded most families' income. Apart from lack of financial support, mothers were not getting support from their husbands, friends, and other relatives. This is confirmed with what was reported by UNICEF where it was observed that extra costs of caring for infants with DDM increases the rate of divorce among families with affected infants.^35^ Infants with DDM may need extensive medical follow up and interventions of specialized therapies such as speech and physical therapy that are often helpful for infant's development. The frequency and duration of these interventions can increase the cost for healthcare and traveling for sessions with therapists, healthcare visits to obtain specific treatments that may not be offered nearby can result in major expenses for families.

Conflicts in marriage due to lack of acceptance of the infant's condition by the parents and husbands were reported to have been blaming and accusing mothers, thinking that the child disability was caused by the mother. This is comparable with another study conducted in Japan which showed that 35% of parents who raise infants with disabilities do not stay together for longer than 10 years. Female parents are, therefore, chased away from the families by their husband, this action may end up exposing such mothers to many challenges in raising affected infants and they may suffer from mental challenges, such as depression.^36^ Taking care of a child with DDM can be emotionally, physically, and financially demanding, and the stresses associated with disabilities may contribute to marriage-related problems.

Sometimes neighbours would talk very badly about the child. They thought that the mother had caused the disability of the child, the situation which is disappointing parents. The above findings are supported by Alaee & Kermanshahi^32^ who reported limitation of parents' social relations, intrapersonal conflicts and shortage of healthcare facilities to manage DDM infants. Lack of awareness about DDM can cause negative perceptions and misconceptions which can result in negative attitudes, whenever there is a lack of accurate information, gossip and rumours can occur.

Stigmatisation is another challenge that was being encountered by parents and which affected infants. The similar study found that mothers of infants with DDM were stigmatized and isolated^17^ because to have a child with DDM is regarded as taboo. The terms used to refer to infants with DDM were creating psychological trauma to parents of such infants. Stigma associated with having infants with DDM made parents feel that giving birth to an infant with DDM was something to be ashamed.^17^ These findings are comparable to the study which indicated that some parents of infants with autism experienced suppressive emotion due to stigmatization. The parents with affected infants are seen to be involved in witchcraft or are believed to be paying for previous committed sins.^37^ Diverse cultural believes and misconceptions with regard to DDM may lead to stigmatization against parents of infants with DDM. The common causes of stigma included lack of understanding, fear and sometimes cultural believes.

### Child Development Activities

Talking, standing and playing games were identified as developmental activities or part of treatment for infants with DDM. As proposed by Werner^38^ that the infants with DDM need developmental activities like talking, playing and singing, asking questions and give the time to answer. Similar to the findings of previous study,^39^ while some parents did not know what to do with their weak infants, in some families, the mother was the only person who was performing those exercises to the child, and in other families, the task was left with the house helper. Because most men are not involved, more mothers than fathers know how to perform child developmental activities for their affected infants.^40^ The strong emotional attachment between mothers and their infants might encourage mothers to become more involved in their child's developmental activities where toys and games were used at home to improve motor development of their child with DDM.

### Study Limitations

This study did not involve other regions which could have added different experience and identify varying challenges faced by parents from different regions. Further studies should be conducted in other regions to explore their experiences and challenges in caring for infants with developmental challenges. However, this study generated evidence that can be used to develop strategies for improving care infants with DDM.

## CONCLUSION

Mothers of children with DDM are stigmatized and accused for giving birth to child with DDM. Lack of special health care facility for children with DDM, high treatment costs, conflict in marriage and lack of spouse, partner, relatives and community support are some of the challenges encountered by mothers of children with DDM.

### Recommendations

To improve quality of life of infants with DDM, parents should be provided with appropriate information about DDM and caring support instead of being stigmatized or accused. Male spouses should be educated on importance of their involvement in caring for child with DDM. To lighten the financial burden of caring for infants with DDM, the government ought to develop a policy that waives medical expenses for infants with DDM.
